# The Relationships between Water Intake and Hydration Biomarkers and the Applications for Assessing Adequate Total Water Intake among Young Adults in Hebei, China

**DOI:** 10.3390/nu13113805

**Published:** 2021-10-26

**Authors:** Jianfen Zhang, Guansheng Ma, Songming Du, Na Zhang

**Affiliations:** 1 Department of Nutrition and Food Hygiene, School of Public Health, Peking University, 38 Xue Yuan Road, Haidian District, Beijing 100191, China; ZJF@bjmu.edu.cn (J.Z.); mags@bjmu.edu.cn (G.M.); 2 Laboratory of Toxicological Research and Risk Assessment for Food Safety, Department of Nutrition and Food Hygiene, School of Public Health, Peking University, 38 Xue Yuan Road, Haidian District, Beijing 100191, China; 3 Chinese Nutrition Society, Room 1405, Beijing Broadcasting Building, No. 14 Jianguomenwai Street, Chaoyang District, Beijing 100053, China; dusm@cnsoc.org

**Keywords:** hydration biomarkers, water intake, relationship, applications

## Abstract

Water is an essential nutrient for humans. A cross-sectional study was conducted among 159 young adults aged 18–23 years in Hebei, China. The total drinking fluids and water from food were obtained by 7-day 24 h fluid intake questionnaires and the duplicate portion method, respectively. Pearson’s correlation coefficients were performed to determine the relationship between fluid intake and 24 h urinary biomarkers and plasma biomarkers. A multivariable partial least squares (PLS) model was used to identify the key predictors in modeling the total water intake (TWI) with 24 h urine biomarkers. Logistic regressions of the TWI against binary variables were performed, and the receiver operating characteristic curve (ROC) was analyzed to determine the cutoff value of the TWI for the optimal hydration status and dehydration without adjustments to favor either the sensitivity or specificity. In total, 156 participants (80 males and 76 females) completed the study. Strong relationships were found between the total drinking fluids, TWI, and 24 h urine biomarkers among young adults, especially for the 24 h urine volume (*r* = 0.784, *p* < 0.001; *r* = 0.747, *p* < 0.001) and osmolality (*r* = −0.589, *p* < 0.001; *r* = −0.477, *p* < 0.001), respectively. As for the FMU and plasma biomarkers, no strong relationships were found. The percentages of the variance in TWI explained by the PLS model with 13 urinary biomarkers were 66.9%. The optimal TWI values for assessing the optimal hydration and dehydration were 2892 mL and 2482 mL for young males, respectively, and 2139 mL and 1507 mL for young females, respectively. Strong relationships were found between the TWI, total drinking fluids, and 24 h urine biomarkers, but not with the FMU and plasma biomarkers, among young adults, including males and females. The 24 h urine biomarkers were more sensitive than the first morning urinary biomarkers in reflecting the fluid intake. The TWI was a reliable index for assessing the hydration statuses for young adults in free-living conditions.

## 1. Introduction

Water is the main constituent of cells, tissues, and organs, and it is the most essential nutrient [1]. Different organs have different contents of water, i.e., 83.0% in the blood, 74.8% in the brain, and 22.0% in the skeletal muscle. The intake and output of the water are in balance, and the loss of 1% of the body water is usually compensated within 24 h. In an appropriate environment with a temperature of 18–25 °C, the amounts of the water losses of a healthy sedentary adult range from 1.8 L to 3.0 L [2]. The intakes of the water must be counterbalanced with the outputs to maintain the stability of the hydration status, as an insufficient intake of water or excessive output of water may lead people into dehydration. The link between water intake and health has recently been highlighted for a variety of health-related outcomes. Studies focusing on the hydration status have shown that chronic or acute dehydration is linked with medical problems, including urological, gastrointestinal, and circulatory disorders [3,4]. Moreover, a series of studies has demonstrated that dehydration has adverse effects on cognitive performances and diminishes some aspects of mood among children, young adults, and elderly people [5,6,7].

It is of vital importance for people to have sufficient water intake to maintain the appropriate hydration status. Furthermore, it is also necessary for people to learn how to judge their hydration status by themselves. There are a few indexes to evaluate the hydration status of humans [8], including acute and chronic dehydration, but no single index is appropriate to assess all the different hydration statuses. For instance, the loss of body mass is suitable to assess acute dehydration [9], but it is not applicable for prolonged studies. Moreover, plasma osmolality reflected intracellular osmolality; therefore, it is considered a most valid marker to reflect the acute changes of hydration status [10]. Regarding the urinary biomarkers, the void of 24 h urine is an indicator for chronic changes of hydration status [11,12]. The urine osmolality was used to determine the hydration status for people in free-living conditions, as well as the urine color [13,14,15,16]. It was showed that the urinary biomarkers varied according to the fluids’ intake, both in China and other countries [17,18]. Studies also demonstrated that the hydration biomarkers, including the urinary and the plasma biomarkers, were significant in terms of predicting health outcomes. For instance, the osmolality of urine indicated the ability of the kidney to concentrate the urine and reflected the antidiuretic action of vasopressin [19,20]. In addition, it also could be an environmental biomonitoring index among people for measuring the exposure [21]. In addition, an association exists between the osmolality of plasma and the mortality among patients undergoing hemodialysis [22]. Therefore, it is crucial to explore the hydration biomarkers among people. Meanwhile, there is conflicting evidence in the literature about some of the abovementioned indexes and their ability to detect hydration status. Therefore, it is crucial to establish more reliable indexes to assess the hydration status. To date, indexes to assess hydration status have been scarcely studied in China.

A series of studies has explored the hydration statuses and total water intake (TWI), including the total drinking fluids and water from food, among different ages of people. A cross-sectional study conducted among young adults revealed that participants with higher total drinking fluids had better hydration status, with lower urine osmolality and higher urine volume than those with lower total drinking fluids. However, no differences were found in plasma biomarkers [17]. Similarly, young females with fluid intake of <1.2 L/d produced a smaller amount of more concentrated urine than participants with 2–4 L/d fluids intake [18]. Studies evaluating the associations between hydration biomarkers and water intake among adults, children, and pregnant and lactating women showed that strong correlations were found between total water intake and urine osmolality and USG [23,24,25,26]. However, few studies have been implemented among people in China, except one study conducted among young males [27], which did not include females. Studies revealed that many factors including the anthropometry, and the environmental such as the temperature and humidity, affected the intakes of the fluid. Furthermore, the drinking patterns including the amounts and types of fluids intake and the food may affect the hydration status of people [28,29]. The osmolality, USG, and color differed significantly among adults from different countries [25]. Thus, although the studies related to the water intake and hydration biomarkers among people had been conducted in some countries, more studies are needed to explore the issue in China to supply more information for the fluids intake and hydration status.

Regarding the adequate intake of water for people, the guidelines for the adequate water intake for people with different ages have been proposed by many countries according to the characteristics of people. The WHO recommends an adequate water intake for males and females of 2.9 L and 2.2 L, respectively [30]. The European Food Safety Authority (EFSA) recommends a daily TWI of 2.5 L for men and 2.0 L for women to maintain urinary osmolality of 500 mOsmol/L [31]. In the United States, the IMO (Institute of Medicine) recommends 3.7 L for males and 2.7 L for females [32]. In China, the recommendations of TWI for men and women are 3.0 L/d and 2.7 L/d, respectively. China’s recommendations are based on one large survey conducted among adults aged 18–60 years in four cities [33]. However, the urine biomarkers were not included in the research, and the association between the water intake and hydration status was scarcely studied. Furthermore, in China, the TWI assessing the optimal hydration status and dehydration were only conducted among young males [27]. However, the TWI assessing the hydration statuses among females were not evaluated. Therefore, this area needs more studies to be implemented.

The aims of the study were (1) to investigate the associations between the fluid intake and the urinary and plasma biomarkers and (2) to explore the TWI for assessing the optimal hydration and dehydration statuses among young males and females.

## 2. Materials and Methods

### 2.1. Participants

The recruitment of the participants was conducted at a college in Baoding, Hebei province. The inclusion criteria were that participants should be aged 18–23 years and healthy. The exclusion criteria included the following: habit of smoking, alcohol consumptions (>20 g/day), or habitual high caffeine consumption (>250 mg/day), or the presence of chronic diseases or other diseases [34]. The recruitment notices were sent through WeChat (Tencent Holdings Ltd., Shenzhen, China) and email. In addition, the advertisements were also posted in the campus publicity window. Furthermore, the participant recruitment campus says that all college students that could participate also received them.

### 2.2. Study Design and Procedure

This was a cross-sectional study which lasted for 7 days.

**Figure 1:**
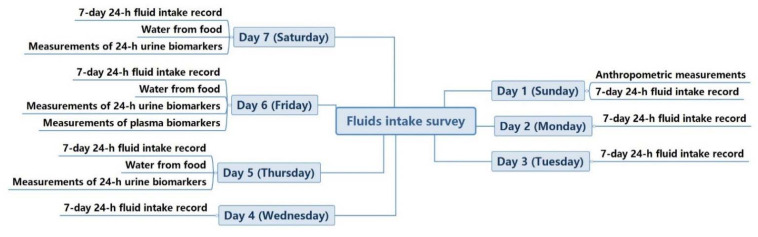
Study procedure.

The cross-sectional study was designed and conducted as described in detail in our previous study [35]. The study period included 7 consecutive days. The participants were asked to complete the 7-day 24 h fluid intake questionnaire by themselves under the supervision of the researchers for 7 consecutive days. On the first day of the study, the anthropometric measurements, including height and weight, were performed. From the fifth day to the seventh day of the study, the participants were asked to collect all urine, and all the food they ate was weighed and recorded during the 3 days. On the sixth day, the fasting blood samples of all participants were collected. The indoor and outdoor temperature and humidity were recorded at 10:00 a.m., 2:00 p.m., and 8:00 p.m. each day for 7 days, as shown in our previous study [17]. The study procedure was shown in Figure 1.

### 2.3. Anthropometric Measurements

Height and weight were measured twice by trained investigators in standard procedure (HDM-300; Huaju, Zhejiang, China). (BMI: weight (kg)/height squared (m^2^)). The values of height and weight are shown as the calculated averages, respectively.

### 2.4. Temperature and Humidity of the Environment

The indoor and outdoor temperature and humidity were recorded by trained investigators every day using a temperature hygrometer (WSB-1-H2, Exasace, Zhengzhou, China). The researcher recorded the temperature and humidity every day at 10:00 a.m., 2:00 p.m., and 8:00 p.m.

### 2.5. Assessment of Total Water Intake

#### 2.5.1. Assessment of Total Drinking Fluids

A self-designed 7-day 24 h fluid intake record questionnaire was used to assess the total drinking fluids. The type and amount of fluids for each intake were measured using a cup to the nearest 5 mL.

#### 2.5.2. Assessment of Water from Food

The water from food was assessed with the duplicate portion method. The samples of food being weighed before and after the participants ate, as well as the backup food samples, were collected for 3 consecutive days. All foods were accurately weighed by the trained investigators using electronic balance (YP20001; SPC; Shanghai, China). Moreover, the backup food samples were stored in refrigerators at +4 °C and sent to the laboratory to be measured within 36 h. The samples of foods were measured according to the National Food Safety Standard GB 5009.3–2016 Determination of Water in Food [36] by a laboratory analyst in the Beijing Institute Nutritional Resource. Parallel samples were taken for each food sample, and the error between the two results was no more than 5%. The water intake from fruits was assessed using the China Food Composition Table (2009) [37].

### 2.6. Urine Biomarkers

The 24 h urine was defined from the second urine of the first day to the first urine of the second day. The 24 h urine samples of 3 consecutive days were collected by participants using self-designed containers of the investigators. All the urine samples were stored at +4 °C before measurement. Every urine sample was collected and tested within 2 h. Urine volume was measured to the nearest 0.1 g using a desktop electronic scale (YP20001, SPC, Shanghai, China).

Urine osmolality was assessed with a freezing point method by the osmotic pressure molar concentration meter (SMC 30C; Tianhe, Tianjin, China).

USG (urine specific gravity) and pH were tested by the automatic urinary sediment analyzer with the uric dry-chemistry method (H-800; Dirui, Changchun, China).

Urine electrolyte concentrations (including Na, K, Cl, Ca, Mg, and phosphate), urine acid, urine urea nitrogen, and creatinine were tested by the automatic biochemical analyzer with the ion-selective electrode potentiometer method (AU 5800; Beckman, Brea, CA, USA).

### 2.7. Plasma Biomarkers

Fasting blood samples were collected for 1 day to measure the osmolality and electrolyte concentrations. Plasma osmolality was assessed with the freezing point method by osmotic pressure molar concentration meter (SMC 30C; Tianhe, Tianjin, China). Blood electrolyte concentrations (including sodium, potassium, chloride, calcium, magnesium, and phosphate) were tested by the automatic biochemical analyzer with the ion-selective electrode potentiometer method (AU 5800; Beckman, Brea, CA, USA).

### 2.8. Statistics

The SAS 9.2 software (SAS Institute Inc., Cary, NC, USA) was used for the statistical analysis. The data with normal distribution or skewness distribution were presented with mean ± standard deviation (SD), median, and quartile ranges (Q), respectively. Pearson’s correlation coefficients were performed to determine the relationship between the fluid intakes and 24 h urinary biomarkers, and between the FMU (first morning urine) and plasma biomarkers. A multivariable partial least squares (PLS) model was used to identify the key predictors in modeling the TWI with 24 h urine biomarkers. A total of 13 urinary biomarkers (urine volume, osmolality, specific gravity, pH, and concentrations of K, Na, Cl, Ca, phosphate, Mg, urea, creatine, and urine acid) were predictors of the TWI. Hydration status was defined according to the osmolality of 24 h urine. The optimal hydration was defined as urine osmolality ≤ 500 mOsm/kg, middle hydration was defined by measurement of 500 mOsm/kg < urine osmolality ≤ 800 mOsm/kg, and dehydration was defined as urine osmolality > 800 mOsm/kg [38]. Binary variables were established based on the 24 h urine osmolality, which was used to assess the optimal hydration (0: optimal hydration; 1: middle hydration + dehydration) and dehydration (0: optimal hydration + middle hydration; 1: dehydration), respectively. Logistic regressions of the TWI against the binary variables were performed, and the receiver operating characteristic curve (ROC) was analyzed to determine the cutoff value of the TWI for the optimal hydration and dehydration status without adjustments to favor either sensitivity or specificity. The significance levels were set at 0.05 (*p* < 0.05).

## 3. Results

A total of 159 participants were recruited for the study. In total, 156 of them completed the study, with a 98% completion rate, including 80 males and 79 females. The age of the males and females was 19.9 years and 19.8 years, respectively. Furthermore, about 23.8% and 51.3% of the males and females were in optimal hydration status. Significant differences were found in the height, weight, BMI, and the hydration statuses among males and females (*p* < 0.05), as shown in Supplementary Table S1.

### 3.1. Temperature and Humidity during the Study

The average indoor and outdoor temperatures for the seven days were 21.8 °C and 20.7 °C, respectively. The average indoor and outdoor humidity were 39.9% and 35.9%, respectively, as shown in Supplementary Table S2.

### 3.2. Association between Fluids Intake and 24 h Urine and First Morning Urine Biomarkers

As shown in Table 1, strong relationships were found between the 24 h urine biomarkers and the total fluid intake and TWI, especially for the 24 h urine volume (*r* = 0.784, *p* < 0.001; *r* = 0.747, *p* < 0.001) and osmolality (*r* = −0.589, *p* < 0.001; *r* = −0.477, *p* < 0.001), respectively. Significant negative correlations between the total drinking fluids and USG, K, Na, Cl, Ca, phosphorus, Mg, urea, uric acid, and creatinine were found (all *p* < 0.001). For the associations between TWI and urinary biomarkers, significant negative correlations were found in USG, K, Ca, phosphorus, Mg, urea, uric acid, and creatinine (all *p* < 0.001). However, for the water from food and urinary biomarkers, weak associations were found, even in the volume of urine (*r* = 0.371, *p* < 0.001). As for the relationships between the FMU biomarkers and total fluids intake and TWI, significant associations were found in the osmolality (*r* = −0.428, *p* < 0.001; *r* = −0.349, *p* < 0.001). Moreover, significant correlations were also found in males (*r* = −0.500, *p* < 0.001; *r* = −0.470, *p* < 0.001) and females (*r* = −0.715, *p* < 0.001; *r* = −0.672, *p* < 0.001), as showed in Supplementary Table S3.

### 3.3. Association between Fluid Intake and Plasma Biomarkers

Except for the weak associations between the total drinking fluids and Ca and Mg (*r* = 0.158, *p* = 0.049; *r* = 2.208, *p =* 0.009), a negative association was found between TWI and phosphorus (*r* = −0.170, *p =* 0.034). No other relationships were found between the plasma biomarkers and the total drinking fluids and TWI, even for the plasma osmolality (*r* = −0.030, *p* = 0.714; *r* = 0.018, *p* = 0.827), as shown in Table 2. There was only one negative association between the water from food and phosphorus (*r* = −0.328, *p* < 0.001), as shown in Table 2. Furthermore, there was only two negative associations between the water from food and phosphorus, magnesium in males (*r* = −0.236, *p* = 0.035; *r* = −0.240, *p* = 0.032); and one negative associations between the water from food and calcium in females (*r* = −0.237, *p =* 0.039), as shown in Supplementary Table S4.

### 3.4. Partial Least Squares Model of the Relationship between TWI and 24 h Urinary Biomarkers

**Figure 2:**
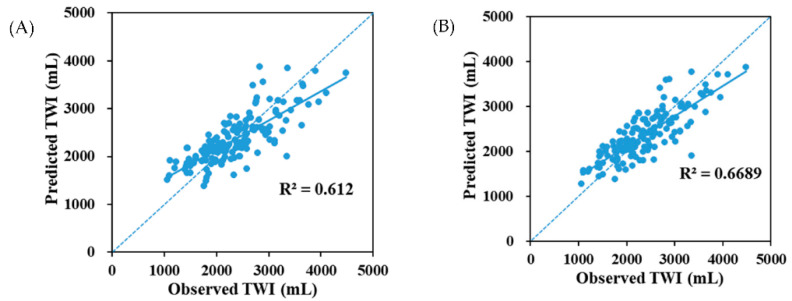
PLS model of the relationship between the total water intake (TWI) and urine biomarkers. (**A**) PLS model for the relationship between TWI and 13 variables. The solid line represents the line agreement, while the dashed line represents the line of best agreement; (**B**) PLS model of the relationship between the TWI and urine volume and urine osmolality. The solid line represents the line agreement, while the dashed line represents the line of best agreement.

A PLS model of the relationship between fluid intake and 24 h urinary biomarkers was developed using 13 urinary measurements as variables. The percentage of variance in TWI (R^2^) explained by the PLS model was 66.89%, with a root mean square error of 390 mL (Figure 2). In the PLS model, two urine biomarkers (24 h urine volume and osmolality) were identified as possible key predictors of the TWI. The 24 h urine volume, Na, Cl, and osmolality primarily contributed to the PLS model, with a variable importance in the projection (VIP) of 2.15, 1.01, 1.06, and 0.86, respectively, as shown in Table 3. In the PLS model with the 24 h urine volume and osmolality as variables, the percentage of variance in the TWI (R^2^) explained by the PLS model was 61.20%, with a root mean square error of 406 mL (Figure 2).

### 3.5. Determination of the TWI for Assessing Optimal Hydration and Assessing Dehydration

**Figure 3:**
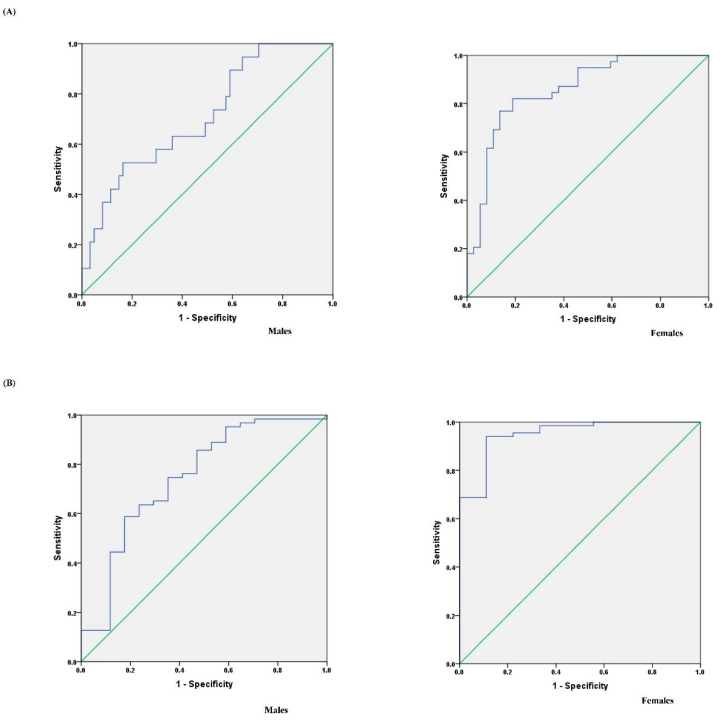
Receiver operating characteristic curve (ROC) analysis curve of the total water intake for assessing optimal hydration and assessing dehydration. (**A**) ROC for assessing optimal hydration in males and females; (**B**) ROC for assessing dehydration in males and females.

The TWI for assessing optimal hydration for males was 2892 mL (area under the curve = 0.712), with a sensitivity of 83.6% and specificity of 52.6%. For females, the TWI for assessing optimal hydration was 2139 mL (area under the curve = 0.857), with a sensitivity of 86.5% and specificity of 76.9%. The TWI for assessing dehydration for males was 2482 mL (area under the curve = 0.745), with a sensitivity of 58.7% and specificity of 82.4%. For females, the TWI for assessing dehydration was 1507 mL (area under the curve = 0.950), with a sensitivity of 94.0% and specificity of 88.9% (Figure 3).

## 4. Discussion

The correlations in our study showed that strong associations were found between the TWI, total drinking fluids, and 24 h urinary biomarkers, respectively, especially the volume and osmolality. However, weak relationships were found between the TWI, total drinking fluids, and first morning urinary biomarkers, respectively, which are consistent with the results found by the authors of [23]. Therefore, we could conclude that the 24 h urinary biomarkers were more sensitive than the first morning urinary biomarkers. Furthermore, as for the relationships between water from food and 24 h urinary biomarkers, no strong associations were found in our study (all *r* < 0.4), which is different than the study conducted among young males. The study conducted among young males showed that the water from food was strongly associated with the volume of urine (*r* = 0.7, *p* < 0.001). After analyzing the associations between the fluids intake and hydration biomarkers, weak correlations between water from food and the volume were found both in males (*r* = 0.412) and females (*r* = 0.493), respectively. Recently, a systematic study showed that the urine productions were affected by multiple dietary factors including the food composition [39], which may be attributed to the differences in our study. Future research is needed to support current evidence and the mechanisms underlying these findings of the present study.

Few associations were found between the TWI, total drinking fluids, and plasma biomarkers, including the osmolality. Therefore, the plasma osmolality was not sensitive to the changes of water intake among young adults in free-living conditions in China. These results are consistent with other studies. A study showed that there were no significant differences in the plasma osmolality between females with higher (2–4 L/d) and lower (<1.2 L/d) water intake [18]. Even in the dehydrated status, which was induced by exercise or fluid deprivation, the plasma osmolality was maintained between 285–290 mOsm/kg [40]. Possibly due to the stability of its vital importance for cardiovascular function, plasma osmolality was not changed in accordance with the urinary biomarkers.

In our study, a PLS model of the relationship between the TWI and 24 h urinary biomarkers was developed. However, the root mean square error was 390 mL, which represents a certain degree of inaccuracy. In a similar model developed among young adults, it was shown that the root mean square error was 663 mL [23], whereas, in China, the root mean square error among the male young adults was 370 mL [27]. In another study implemented among healthy adults, it was suggested that the 24 h urine osmolality is a physiological index of adequate fluid intake [38].

In this study, logistic regression of the fluid intake against a binary hydration outcome was performed. The results showed that the TWI for assessing the optimal hydration (urine osmolality < 500 mOsm/kg) and dehydration (urine osmolality >800 mOsm/kg) in males were 2892 mL and 2482 mL, respectively, which were similar to the results of other studies. In a sample of 59 young males, the well-hydrated status (urine osmolality between 476–586 mOsm/kg) was represented by the means values of TWI between 2454–2614 mL, and the slightly dehydrated status (urine osmolality between 767–880 mOsm/kg) was represented by the mean values of TWI between 2009 and 2048 mL [41]. For females, the TWI for assessing the optimal hydration status (urine osmolality < 500 mOsm/kg) and dehydration (urine osmolality > 800 mOsm/kg) in females were 2139 mL and 1507 mL, respectively. A cross-sectional designed study implemented among young females found similar findings to our study. It showed that the dehydrated status, defined as urine osmolality between 549–705, corresponded to the TWI between 2109–2506 mL. Moreover, the increasing dehydration status, defined as urine osmolality > 810 mOsm/kg, corresponded to the TWI < 1744 mL [42]. It could be concluded that the TWI is a convenient and reliable index to assess the hydration status among humans in the free-living young adults in China.

Our study has some strengths and weaknesses. First, the total drinking fluids were assessed by the 7-day 24 h fluid intake questionnaire, which included the details of the fluid intake, and resulted in a higher estimate of the TWI [43]. Moreover, the water from food was assessed by a duplicate portion method, which made the intake of water from food more accurate and avoided the record bias. Along with the strengths above, our study also had some weaknesses. First, participants from a wide range of age groups were not investigated. Second, more plasma biomarkers, such as copeptin, were not explored. Thirdly, the participants were recruited among one college, and the free-living conditions may be different from other adults not in the campus; therefore, in the future, more studies conducted among young adults of different occupations or characteristics should be put into this issue.

## 5. Conclusions

Strong relationships were found between the TWI and 24 h urine biomarkers, but not with the plasma biomarkers. The 24 h urine biomarkers were more sensitive than the first morning urinary biomarkers in reflecting the fluid intake. The TWI is a reliable index for assessing the hydration statuses for young adults in free-living conditions.

## Figures and Tables

**Table 1:** Correlations between the fluid intake and urinary biomarkers of young adults.

Urine Biomarkers	Total Drinking Fluids		Water from Food		TWI	
	*r*	*p*	*r*	*p*	*r*	*p*
24 h Volume (mL)	0.784	<0.001	0.371	<0.001	0.747	<0.001
Osmolality (mOsm/kg)	−0.589	<0.001	−0.079	0.329	−0.477	<0.001
USG	−0.397	<0.001	−0.098	0.225	−0.370	<0.001
pH	−0.046	0.571	0.102	0.206	0.027	0.741
Potassium (mmol/L)	−0.404	<0.001	−0.036	0.659	−0.353	<0.001
Sodium (mmol/L)	−0.314	<0.001	0.235	0.003	−0.151	0.059
Chloride (mmol/L)	−0.303	<0.001	0.255	0.001	−0.137	0.088
Calcium (mmol/L)	−0.280	<0.001	−0.093	0.251	−0.257	0.001
Phosphorus (mmol/L)	−0.397	<0.001	−0.172	0.032	−0.410	<0.001
Magnesium (mmol/L)	−0.405	<0.001	−0.173	0.031	−0.409	<0.001
Urea (mmol/L)	−0.375	<0.001	−0.090	0.263	−0.351	<0.001
Uric acid (mmol/L)	−0.482	<0.001	−0.143	0.075	−0.458	<0.001
Creatinine (mmol/L)	−0.369	<0.001	−0.135	0.094	−0.373	<0.001
**FMU**						
Osmolality (mOsm/kg)	−0.428	<0.001	−0.009	0.916	−0.349	<0.001
USG	−0.324	<0.001	−0.015	0.855	−0.287	<0.001
pH	−0.072	0.374	0.046	0.571	−0.020	0.806
Potassium (mmol/L)	−0.380	<0.001	−0.011	0.892	−0.327	<0.001
Sodium (mmol/L)	−0.339	<0.001	0.154	0.055	−0.207	0.009
Chloride (mmol/L)	−0.328	<0.001	0.194	0.015	−0.186	0.020
Calcium (mmol/L)	−0.155	0.054	−0.064	0.425	−0.152	0.059
Phosphorus (mmol/L)	−0.352	<0.001	−0.220	0.006	−0.387	<0.001
Magnesium (mmol/L)	−0.226	0.004	−0.105	0.191	−0.238	0.003
Urea (mmol/L)	−0.358	<0.001	−0.068	0.401	−0.325	<0.001
Uric acid (mmol/L)	−0.456	<0.001	−0.153	0.056	−0.430	<0.001
Creatinine (mmol/L)	−0.271	0.001	−0.060	0.460	−0.259	0.001

**Table 2:** Correlations between the fluid intake and plasma biomarkers of young adults.

Plasma Biomarkers	Total Drinking Fluids		Water from Food		TWI	
	*r*	*p*	*r*	*p*	*r*	*p*
Osmolality (mOsm/kg)	−0.030	0.714	0.011	0.889	0.018	0.827
Potassium (mmol/L)	0.045	0.577	0.024	0.769	0.055	0.497
Sodium (mmol/L)	0.124	0.122	0.101	0.173	0.130	0.106
Chloride (mmol/L)	0.014	0.859	0.110	0.173	0.042	0.606
Calcium (mmol/L)	0.158	0.049	0.037	0.646	0.141	0.079
Phosphorus (mmol/L)	−0.049	0.543	−0.328	0.000	−0.170	0.034
Magnesium (mmol/L)	0.208	0.009	−0.095	0.239	0.108	0.178

**Table 3:** Variable importance in the projection (VIP) coefficients for the 24 h urine hydration biomarkers in the partial least squares (PLS) model (TWI).

VIP > 0.8		VIP < 0.8	
Volume	2.15	pH	0.68
Osmolality	0.86	USG	0.79
Sodium (mmol/L)	1.01	Potassium (mmol/L)	0.71
Chloride (mmol/L)	1.06	Calcium (mmol/L)	0.77
Phosphorus (mmol/L)	0.81	Magnesium (mmol/L)	0.72
Creatinine (mmol/L)	0.84	Uric acid (mmol/L)	0.77
Urea (mmol/L)	0.91		

## Data Availability

The data of this study is available from the corresponding author on reasonable request.

## Supplementary Materials

The following are available online at https://www.mdpi.com/article/10.3390/nu13113805/s1, Table S1: The characteristics of young adults, Table S2: The temperature and humidity during the study, Table S3: Correlations between fluids intake and urinary biomarkers of young adults, Table S4: Correlations between fluids intake and plasma biomarkers of young adults.

## References

[1] Popkin B.M., D’Anci K.E., Rosenberg I.H. (2010). Water, hydration, and health. Nutr. Rev..

[2] Grandjean A.C., Reimers K.J., Buyckx M.E. (2003). Hydration: Issues for the 21st century. Nutr. Rev..

[3] El-Sharkawy A.M., Sahota O., Lobo D.N. (2015). Acute and chronic effects of hydration status on health. Nutr. Rev..

[4] Armstrong L.E. (2012). Challenges of linking chronic dehydration and fluid consumption to health outcomes. Nutr. Rev..

[5] Zhang N., Du S.M., Zhang J.F., Ma G.S. (2019). Effects of Dehydration and Rehydration on Cognitive Performance and Mood among Male College Students in Cangzhou, China: A Self-Controlled Trial. Int. J. Environ. Res. Public Health.

[6] Carlson L.A., Lawrence M.A., Kenefick R.W. (2018). Hydration Status and Thermoregulatory Responses in Drivers During Competitive Racing. J. Strength Cond. Res..

[7] Bar-David Y., Urkin J., Kozminsky E. (2005). The effect of voluntary dehydration on cognitive functions of elementary school children. Acta Paediatr..

[8] Baron S., Courbebaisse M., Lepicard E.M., Friedlander G. (2015). Assessment of hydration status in a large population. Br. J. Nutr..

[9] Harvey G., Meir R., Brooks L., Holloway K. (2008). The use of body mass changes as a practical measure of dehydration in team sports. J. Sci. Med. Sport.

[10] Ganio M.S., Armstrong L.E., Casa D.J., McDermott B.P., Lee E.C., Yamamoto L.M., Marzano S., Lopez R.M., Jimenez L., Le Bellego L. (2011). Mild dehydration impairs cognitive performance and mood of men. Br. J. Nutr..

[11] Tucker M.A., Gonzalez M.A., Adams J.D., Burchfield J.M., Moyen N.E., Robinson F.B., Schreiber B.A., Ganio M.S. (2016). Reliability of 24-h void frequency as an index of hydration status when euhydrated and hypohydrated. Eur. J. Clin. Nutr..

[12] Burchfield J.M., Ganio M.S., Kavouras S.A., Adams J.D., Gonzalez M.A., Ridings C.B., Moyen N.E., Tucker M.A. (2015). 24-h Void number as an indicator of hydration status. Eur. J. Clin. Nutr..

[13] Perrier E.T., Johnson E.C., McKenzie A.L., Ellis L.A., Armstrong L.E. (2016). Urine colour change as an indicator of change in daily water intake: A quantitative analysis. Eur. J. Nutr..

[14] McKenzie A.L., Munoz C.X., Ellis L.A., Perrier E.T., Guelinckx I., Klein A., Kavouras S.A., Armstrong L.E. (2017). Urine color as an indicator of urine concentration in pregnant and lactating women. Eur. J. Nutr..

[15] Zhang N., Du S., Zheng M., Tang Z., Yan R., Zhu Y., Ma G. (2017). Urine color for assessment of dehydration among college men students in Hebei, China—A cross-sectional study. Asia Pac. J. Clin. Nutr..

[16] Kavouras S.A., Johnson E.C., Bougatsas D., Arnaoutis G., Panagiotakos D.B., Perrier E., Klein A. (2016). Validation of a urine color scale for assessment of urine osmolality in healthy children. Eur. J. Nutr..

[17] Zhang J., Zhang N., Wang Y., Liang S., Liu S., Du S., Xu Y., He H., Cai H., Ma G. (2020). Drinking patterns and hydration biomarkers among young adults with different levels of habitual total drinking fluids intake in Baoding, Hebei Province, China: A cross-sectional study. BMC Public Health.

[18] Perrier E., Vergne S., Klein A., Poupin M., Rondeau P., Bellego L.L., Armstrong L.E., Lang F., Stookey J., Tack I. (2013). Hydration biomarkers in free-living adults with different levels of habitual fluid consumption. Br. J. Nutr..

[19] Torres V.E., Grantham J.J., Chapman A.B., Mrug M., Bae K.T., King B.F., Wetzel L.H., Martin D., Lockhart M.E., Bennett W.M. (2011). Consortium for Radiologic Imaging Studies of Polycystic Kidney Disease (CRISP). Potentially modifiable factors affecting the progression of autosomal dominant polycystic kidney disease. Clin. J. Am. Soc. Nephrol..

[20] Lee M.J., Chang T.I., Lee J., Kim Y.H., Han S.H. (2019). Urine Osmolality and Renal Outcome in Patients with Chronic Kidney Disease: Results from the KNOW-CKD. Kidney Blood Press R.

[21] Yeh H.C., Lin Y.S., Kuo C.C., Weidemann D., Weaver V., Fadrowski J., Neu A., Navas-Acien A. (2015). Urine osmolality in the US population: Implications for environmental biomonitoring. Environ. Res..

[22] Tsujimoto Y., Tsutsumi Y., Ohnishi T., Kimachi M., Yamamoto Y., Fukuhara S. (2020). Low Predialysis Plasma Calculated Osmolality Is Associated with Higher All-Cause Mortality: The Japanese Dialysis Outcomes and Practice Patterns Study (J-DOPPS). Nephron.

[23] Perrier E., Rondeau P., Poupin M., Le Bellego L., Armstrong L.E., Lang F., Stookey J., Tack I., Vergne S., Klein A. (2013). Relation between urinary hydration biomarkers and total fluid intake in healthy adults. Eur. J. Clin. Nutr..

[24] McKenzie A.L., Perrier E.T., Guelinckx I., Kavouras S.A., Aerni G., Lee E.C., Volek J.S., Maresh C.M., Armstrong L.E. (2017). Relationships between hydration biomarkers and total fluid intake in pregnant and lactating women. Eur. J. Nutr..

[25] Malisova O., Athanasatou A., Pepa A., Husemann M., Domnik K., Braun H., Mora-Rodriguez R., Ortega J.F., Fernandez-Elias V.E., Kapsokefalou M. (2016). Water Intake and Hydration Indices in Healthy European Adults: The European Hydration Research Study (EHRS). Nutrients.

[26] Kavouras S.A., Bougatsas D., Johnson E.C., Arnaoutis G., Tsipouridi S., Panagiotakos D.B. (2017). Water intake and urinary hydration biomarkers in children. Eur. J. Clin. Nutr..

[27] Zhang N., Du S., Tang Z., Zheng M., Yan R., Zhu Y., Ma G. (2017). Hydration, Fluid Intake, and Related Urine Biomarkers among Male College Students in Cangzhou, China: A Cross-Sectional Study—Applications for Assessing Fluid Intake and Adequate Water Intake. Int. J. Environ. Res. Public Health.

[28] Bougatsas D., Arnaoutis G., Panagiotakos D.B., Seal A.D., Johnson E.C., Bottin J.H., Tsipouridi S., Kavouras S.A. (2018). Fluid consumption pattern and hydration among 8-14 years-old children. Eur. J. Clin. Nutr..

[29] Kenney E.L., Long M.W., Cradock A.L., Gortmaker S.L. (2015). Prevalence of inadequate hydration among US children and disparities by gender and race/ethnicity: National Health and Nutrition Examination Survey, 2009–2012. Am. J. Public Health.

[30] World Health Organization (2005). Nutrients in Drinking Water.

[31] European Food Safety Authority (2010). Scientific Opinion on Dietary Reference Values for water. EFSA J..

[32] Institute of Medicine (US) (2005). DRI, Dietary Reference Intakes for Water, Potassium, Sodium, Chloride, and Sulfate.

[33] Ma G., Zhang Q., Liu A., Zuo J., Zhang W., Zou S., Li X., Lu L., Pan H., Hu X. (2012). Fluid intake of adults in four Chinese cities. Nutr. Rev..

[34] Pross N., Demazieres A., Girard N., Barnouin R., Metzger D., Klein A., Perrier E., Guelinckx I. (2014). Effects of changes in water intake on mood of high and low drinkers. PLoS ONE.

[35] Zhang J., Zhang N., Liang S., Wang Y., Liu S., Liu S., Du S., He H., Xu Y., Cai H. (2018). The amounts and contributions of total drinking fluids and water from food to total water intake of young adults in Baoding, China. Eur. J. Nutr..

[36] Standardization Administration of China (2016). National Food Safety Standard GB5009.3–2016. Determination of Moisture in Foods.

[37] Institute for Nutrition and Health, Chinese Center for Disease Control and Prevention (2009). China Food Composition.

[38] Perrier E.T., Buendia-Jimenez I., Vecchio M., Armstrong L.E., Tack I., Klein A. (2015). Twenty-four-hour urine osmolality as a physiological index of adequate water intake. Dis. Markers.

[39] Alwis U.S., Haddad R., Monaghan T.F., Abrams P., Dmochowski R., Bower W., Wein A.J., Roggeman S., Weiss J.P., Mourad S. (2020). Impact of food and drinks on urine production: A systematic review. Int. J. Clin. Prac..

[40] Szinnai G., Schachinger H., Arnaud M.J., Linder L., Keller U. (2005). Effect of water deprivation on cognitive-motor performance in healthy men and women. Am. J. Physiol. Regul. Integr. Comp. Physiol..

[41] Armstrong L.E., Pumerantz A.C., Fiala K.A., Roti M.W., Kavouras S.A., Casa D.J., Maresh C.M. (2010). Human hydration indices: Acute and longitudinal reference values. Int. J. Sport Nutr. Exerc. Metab..

[42] Armstrong L.E., Johnson E.C., Munoz C.X., Swokla B., Le Bellego L., Jimenez L., Casa D.J., Maresh C.M. (2012). Hydration biomarkers and dietary fluid consumption of women. J. Acad. Nutr. Diet..

[43] Gandy J., Martinez H., Guelinckx I., Moreno L.A., Bardosono S., Salas-Salvado J., Kavouras S.A. (2016). Relevance of Assessment Methods for Fluid Intake. Ann. Nutr. Metab..

