# Supplementary material for: The Relationships between Water Intake and Hydration Biomarkers and the Applications for Assessing Adequate Total Water Intake among Young Adults in Hebei, China
Source: Nutrients. 2021 Oct 26;13(11):3805. doi: 10.3390/nu13113805 (PMC8623709; doi:10.3390/nu13113805)
Supplement: Supplementary file 1 [file nutrients-13-03805-s001.zip › nutrients-1401720-supplementary.pdf]

Supplementary Table S1 The characteristics of young adults

	Male	Female	Total	*P*
Age (y)	19.9±1.0	19.8±1.1	19.8±1.1	0.363
Height (cm)	172.0±5.5	160.1±6.1	166.2±8.3	＜0.001
Weight (kg)	67.5±11.9	54.8±6.6	61.3±11.5	＜0.001
BMI (kg/m^2)	22.8±3.9	21.4±2.2	22.1±3.3	＜0.001
Hydration status				
Optimal hydration	19 (23.8%)	39 (51.3%)	58 (37.1%)	0.002*
Middle hydration	44 (55.0%)	28 (36.8%)	72 (46.2%)	
Dehydration	17 (21.3%)	9 (11.8%)	26 (16.7%)	

Note: *,(χ^2=12.820)

Supplementary Table 2 The temperature and humidity during the study

	Indoors		Outdoors	
	Temperature (℃)	Humidity (%)	Temperature (℃)	Humidity (%)
Sunday	19.9	43	18.1	37
Monday	23.0	48	22.4	41
Tuesday	23.3	31	24.0	29
Wednesday	21.5	48	17.9	42
Thursday	21.5	40	21.0	36
Friday	22.2	35	19.2	35
Saturday	21.2	34	22.6	31

Supplementary Table 3 Correlations between fluids intake and urinary biomarkers of young adults

	Males						Females					
	Total drinking fluids		Water from food		TWI		Total drinking fluids		Water from food		TWI	
**Urine biomarkers**	*r*	*p*	*r*	*p*	*r*	*p*	*r*	*p*	*r*	*p*	*r*	*p*
24h Volume (mL)	**0.743**	<0.001	**0.412**	<0.001	**0.737**	<0.001	**0.832**	<0.001	**0.493**	<0.001	**0.880**	<0.001
Osmolality (mOsm/kg)	**-0.600**	<0.001	-0.158	0.162	**-0.514**	<0.001	**-0.822**	<0.001	**-0.348**	0.002	**-0.799**	<0.001
USG	**-0.432**	<0.001	-0.191	0.090	**-0.425**	<0.001	**-0.683**	<0.001	**-0.472**	<0.001	**-0.738**	<0.001
pH	0.020	0.857	0.191	0.089	0.110	0.331	-0.072	0.534	**0.267**	0.020	0.063	0.587
Potassium (mmol/L)	**-0.384**	<0.001	-0.052	0.648	**-0.312**	0.005	**-0.652**	0.001	**-0.287**	0.012	**-0.641**	<0.001
Sodium (mmol/L)	**-0.222**	<0.001	**0.254**	0.023	-0.034	0.762	**-0.719**	<0.001	-0.201	0.082	**-0.661**	<0.001
Chloride (mmol/L)	**-0.233**	<0.001	**0.287**	0.010	-0.030	0.795	**-0.695**	<0.001	-0.181	0.117	**-0.638**	<0.001
Calcium (mmol/L)	**-0.315**	<0.001	**-0.251**	0.025	**-0.380**	0.001	**-0.444**	<0.001	**-0.268**	0.019	**-0.452**	<0.001
Phosphorus (mmol/L)	**-0.441**	<0.001	**-0.341**	0.002	**-0.508**	<0.001	**-0.702**	<0.001	**-0.526**	<0.001	**-0.780**	<0.001
Magnesium (mmol/L)	**-0.378**	<0.001	**-0.206**	0.067	**-0.389**	<0.001	**-0.629**	<0.001	**-0.384**	0.001	**-0.667**	<0.001
Urea (mmol/L)	**-0.422**	<0.001	**-0.315**	0.004	**-0.464**	<0.001	**-0.755**	<0.001	**-0.465**	<0.001	**-0.790**	<0.001
Uric acid (mmol/L)	**-0.438**	<0.001	**-0.276**	0.013	**-0.483**	<0.001	**-0.818**	<0.001	**-0.414**	<0.001	**-0.822**	<0.001
Creatinine (mmol/L)	**-0.438**	<0.001	**-0.443**	<0.001	**-0.562**	<0.001	**-0.798**	<0.001	**-0.505**	<0.001	**-0.839**	<0.001
**FMU**	*r*	*p*	*r*	*p*	*r*	*p*	*r*	*p*	*r*	*p*	*r*	*p*
Osmolality (mOsm/kg)	**-0.500**	<0.001	-0.196	0.081	**-0.470**	<0.001	**-0.715**	<0.001	**-0.230**	0.045	**-0.672**	<0.001
USG	**-0.349**	0.002	-0.187	0.097	**-0.350**	0.001	**-0.542**	<0.001	-0.156	0.179	**-0.531**	<0.001
pH	0.092	0.419	0.147	0.301	0.122	0.282	**-0.227**	0.049	-0.196	0.091	**-0.239**	0.038
Potassium (mmol/L)	**-0.309**	0.005	-0.041	0.720	**-0.256**	0.022	**-0.548**	<0.001	-0.109	0.348	**-0.504**	<0.001
Sodium (mmol/L)	**-0.275**	0.014	0.129	0.253	**-0.155**	0.169	**-0.724**	<0.001	-0.163	0.160	**-0.673**	<0.001
Chloride (mmol/L)	**-0.309**	0.005	**0.149**	0.188	**-0.173**	0.125	**-0.690**	<0.001	-0.079	0.500	**-0.621**	<0.001
Calcium (mmol/L)	-0.218	0.052	-0.162	0.151	-0.238	0.033	**-0.364**	<0.001	**-0.237**	0.039	**-0.371**	<0.001
Phosphorus (mmol/L)	**-0.251**	0.025	**-0.404**	<0.001	**-0.370**	0.001	**-0.518**	<0.001	**-0.244**	0.034	**-0.508**	<0.001

Magnesium (mmol/L)	**-0.158**	0.162	-0.169	0.135	**-0.195**	0.083	**-0.508**	<0.001	**-0.293**	0.010	**-0.553**	<0.001
Urea (mmol/L)	**-0.373**	0.001	**-0.322**	0.004	**-0.428**	<0.001	**-0.692**	<0.001	**-0.272**	0.017	**-0.659**	<0.001
Uric acid (mmol/L)	**-0.355**	0.001	**-0.220**	0.050	**-0.369**	0.001	**-0.695**	<0.001	**-0.268**	0.019	**-0.677**	<0.001
Creatinine (mmol/L)	**-0.295**	0.008	**-0.379**	0.001	**-0.392**	<0.001	**-0.530**	<0.001	-0.167	0.149	**-0.503**	<0.001

Supplementary Table 4 Correlations between fluids intake and plasma biomarkers of young adults

**Plasma biomarkers**	Total drinking fluids		Water from food		TWI	
	r	*p*	*r*	*p*	*r*	*p*
Males						
Osmolality (mOsm/kg)	-0.037	0.742	-0.008	0.947	-0.014	0.904
Potassium (mmol/L)	0.078	0.494	-0.018	0.876	0.028	0.804
Sodium (mmol/L)	-0.051	0.656	-0.093	0.412	-0.093	0.411
Chloride (mmol/L)	0.123	0.279	0.058	0.612	0.136	0.229
Calcium (mmol/L)	0.105	0.353	0.037	0.745	0.106	0.349
Phosphorus (mmol/L)	-0.122	0.281	**-0.236**	0.035	-0.178	0.113
Magnesium (mmol/L)	0.153	0.175	**-0.240**	0.032	-0.006	0.960
Females						
Osmolality (mOsm/kg)	0.033	0.779	0.060	0.604	0.076	0.513
Potassium (mmol/L)	0.027	0.817	0.054	0.644	0.048	0.680
Sodium (mmol/L)	0.162	0.163	0.074	0.526	0.171	0.139
Chloride (mmol/L)	-0.064	0.584	0.184	0.111	-0.009	0.941
Calcium (mmol/L)	0.078	0.506	**-0.237**	0.039	0.009	0.936
Phosphorus (mmol/L)	0.190	0.100	-0.094	0.421	0.127	0.274
Magnesium (mmol/L)	0.154	0.183	-0.207	0.072	0.056	0.634
